# The double posteromedial portals endoscopy for posterior ankle impingement syndrome in athletes

**DOI:** 10.1186/s10195-022-00651-w

**Published:** 2022-07-06

**Authors:** Nicola Maffulli, Rocco Aicale, Filippo Migliorini, Emilio Wagner, Amol Saxena, Francesco Oliva

**Affiliations:** 1 Department of Musculoskeletal Disorders, Faculty of Medicine and Surgery, University of Salerno, 84084 Baronissi, Italy; 2 Clinica Ortopedica, Ospedale San Giovanni Di Dio E Ruggi D’Aragona, 84131 Salerno, Italy; 3 Barts and the London School of Medicine and Dentistry, Centre for Sports and Exercise Medicine, Queen Mary University of London, Mile End Hospital, 275 Bancroft Road, London, E1 4DG England; 4 Faculty of Medicine, School of Pharmacy and Bioengineering, Guy Hilton Research Centre, Keele University, Thornburrow Drive, Hartshill, Stoke-on-Trent, ST4 7QB England; 5 Department of Orthopaedics, Trauma, and Reconstructive Surgery, RWTH University Hospital, RWTH Aachen University Clinic, Pauwelsstraße 30, 51074 Aachen, Germany; 6 Department of Foot and Surgery, Universidad del Desarrollo, Clínica Alemana, Santiago, Chile; 7 Department of Sports Medicine, Sutter-Palo Alto, Palo Alto, CA United States

**Keywords:** Ankle impingement, Os trigonum, PAIS, Posterior ankle endoscopy

## Abstract

**Background:**

Posterior ankle impingement syndrome (PAIS) may result from flexor hallucis longus tendinopathy, compression of the posterior process of the talus from the presence of an os trigonum, soft-tissue impingement, or a combination of these. Posterior extra-articular endoscopy performed with the patient supine through the double posteromedial portals, with excision of adhesions, excision of the posterior process of the talus or an os trigonum, and decompression of the tendon of the flexor hallucis longus (FHL), can be used in athletes with PAIS.

**Methods:**

Thirty-four athletes with PAIS in whom conservative management had failed underwent posterior ankle endoscopy in the supine position using the double posteromedial portals. The patients were assessed pre- and postoperatively using the American Orthopaedic Foot and Ankle Society hindfoot scale score, the Tegner scale, and the simple visual analogue scale. Time of surgery, return to sports, patient satisfaction, and complications were recorded and analysed. The average length of postoperative follow-up was 26.7 ± 12.6 (range 24 to 72) months.

**Results:**

The mean Tegner activity scale score improved to 9 ± 0.2 postoperatively (*p* < 0.05), while the mean American Orthopaedic Foot and Ankle Society scale score improved to 96 ± 5.1 (range 87 to 100) postoperatively, with 29 of 34 patients (85.3%) achieving a perfect score of 100 (*p* < 0.05). The mean time to return to sports was 8.7 ± 0.7 (range 8 to 10) weeks. The complication rate was low, with no superficial wound infections or venous thromboembolism events; only two patients (5.9%) reported pain and tenderness by 3 months after the index procedure.

**Conclusion:**

Posterior ankle endoscopy for the resection of a posterior process of the talus or an os trigonum and decompression of the tendon of FHL is safe and allows excellent outcomes with low morbidity in athletes with PAIS.

## Introduction

Posterior ankle impingement syndrome (PAIS) is characterised by compression in the anatomic region between the posterior tibia and calcaneus during plantar flexion. PAIS can result from flexor hallucis longus tendinopathy, pain from compression of the posterior process of the talus or the os trigonum, soft-tissue impingement, or a combination of these. PAIS can be encountered in athletes who require forced plantar flexion of the ankle [1]. Surgical management for PAIS was first described by Howse in 1982, who operated using posterior block of the ankle joint in a population of elite dancers [2], naming the condition “talar compression syndrome” [3].

Patients with PAIS report posterior lower ankle pain especially during forced plantar flexion [1], such as during soccer, running, martial arts, fighting sports, and dancing [4], presumably from repetitive weight bearing in maximum plantar flexion [5, 6]. Appropriate plain lateral radiographs with 25° external rotation reveal posterior bony abnormalities, including the Stieda process or an os trigonum, which can support the clinical diagnosis [7]. Magnetic resonance imaging (MRI) can be undertaken when the diagnosis is unclear, allowing evaluations of bone edema, joint effusion, synovitis, tenosynovitis, and chondral injury. Ultrasound (US) has recently gained popularity, as it can reliably and inexpensively aid in identifying the anatomical bases of PAIS [8, 9], and it allows the administration of both diagnostic and therapeutic injections [10–12].

Nonsurgical management remains the initial approach to PAIS, and, for acute symptoms, a period of rest and protection are recommended. Conservative management—including rest, ice, the use of nonsteroidal drugs (NSAIDS), and avoidance of provocative activities—can be successful, together with shoe modifications, including heel lift orthoses to prevent dorsiflexion [13].

When nonoperative management fails and symptoms impact activities of daily living or sport performance, surgery may be indicated [14–16]. A systematic review including 47 articles with a total of 905 patients managed surgically with endoscopic or open approaches for PAIS showed a significantly lower complication rate (7.2% vs. 15.9%, respectively) and an earlier return to full activity (11.3 vs. 16 weeks, respectively) in patients treated endoscopically [17]. Both the open procedure and the endoscopic approach yielded acceptable outcomes in terms of function and pain. However, complication rates were much lower with endoscopic treatment, and the time taken to return to full activities was much shorter [18].

The present study reports the clinical results of surgery in athletic patients with PAIS using the double posteromedial portal with the patient supine. We hypothesised that the procedure would be safe, reliable, and produce good results in athletic patients with PAIS resistant to conservative management.

## Materials and methods

We prospectively followed a total of 34 consecutive athletes operated on from January 2010 to December 2015 following the failure of conservative management for PAIS. The hospital ethics committee approved this study, and all patients gave written consent before medical procedures. The inclusion criteria of patients included in the present investigation were being an athlete, no previous surgery in the index or contralateral leg, and failure of conservative management for at least 3 months (Table 1).

**Table 1:** Demographic data

Variable	Value
Patients (*n*)	34
Mean age at surgery (years) ± standard deviation, range	26.7 ± 9.0, 15 to 47
Female sex (*n*)	13
Right side (*n*)	21
Mean follow-up time (months) ± standard deviation, range	26.7 ± 12.6, 24 to 72
Mean symptom duration (months) ± standard deviation, range	10.5 ± 2.4, 7 to 14
Sport distribution	
Football	9
Martial arts/combat sports	5
Dancing	8
Gymnastics	5
Basketball	4
Crossfit	3

All patients were secondary and tertiary referrals to the senior author, a fellowship-trained surgeon with 10 years of experience in posterior ankle soft-tissue endoscopy before the start of the study. All patients had experienced signs and symptoms of PAIS for at least 3 months before a positive diagnosis had been formulated. On physical examination, all athletes presented a positive hyperplantarflexion test, with marked pain over the posterolateral or posteromedial side of the ankle and anteriorly to the Achilles tendon. The institutional review board (IRB) was granted for the present study.

Patients were evaluated pre- and postoperatively utilising the American Orthopaedic Foot and Ankle Society (AOFAS) [19] hindfoot scale score, the Tegner movement scale score [20], and the visual analogue scale (VAS) [21]. Recovery time, time to return to sports, and patient satisfaction were recorded.

### Surgical technique

**Fig. 1:**
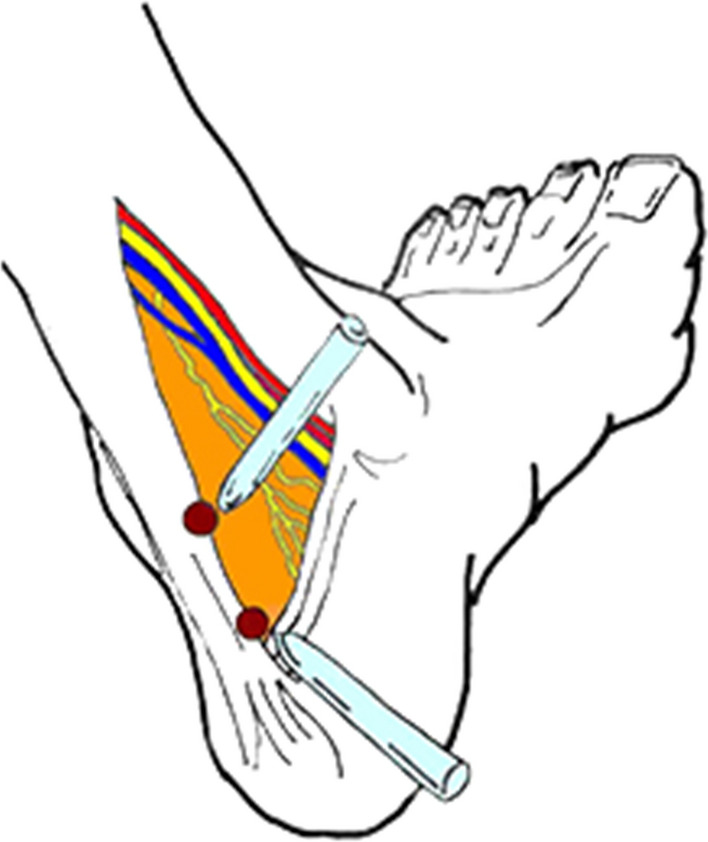
Position of the 2 arthroscopic portals, 45 to 50 mm from each other

**Fig. 2:**
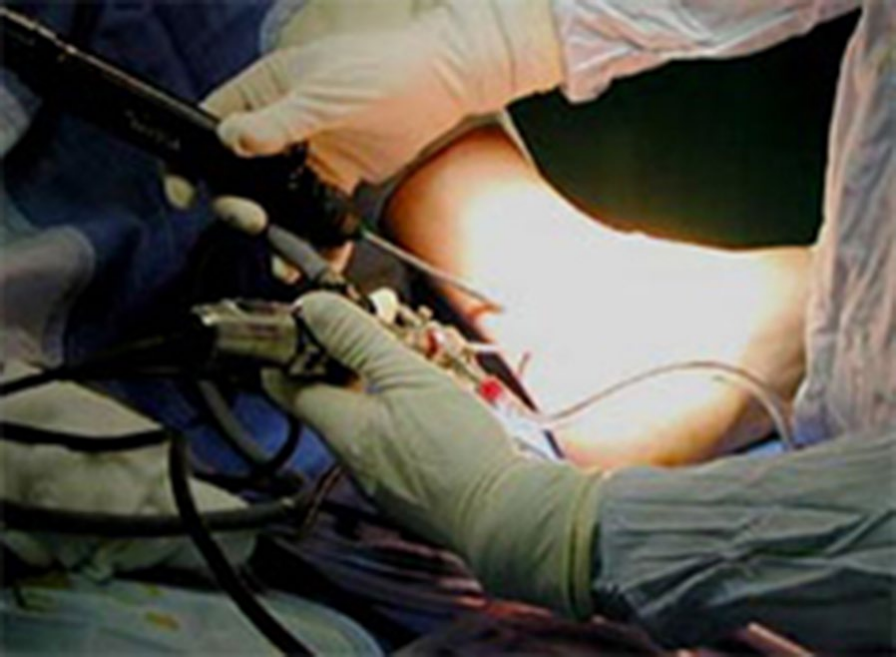
Intra-operative image of portals during surgery

With the patient supine and under general or spinal anaesthesia, a tourniquet was applied on the calf, with its upper edge 2.5 cm distal to the neck of the fibula. The ankle and the foot were felt free of the end of the surgical table, allowing full dorsiflexion of the operated ankle if needed. The leg to be operated was placed in the figure of four position, and the foot was dorsiflexed. The nick and spread technique was used to produce two posteromedial arthroscopic portals just anterior to the anterior margin of the Achilles tendon, 45–50 mm from each other. The first portal was distal, just medial, and anterior to the Achilles tendon, along a horizontal line parallel to the calcaneal tuberosity from the tip of the medial malleolus [16]. The second portal was 45–50 mm proximal to the first one, again just medial and anterior to the Achilles tendon [16] (Fig. 1, 2).

A working area was produced, progressively shaving away the Kager’s fat, proceeding in a posteroanterior and lateral-medial direction from cranial to caudal until the FHL tendon was visualised. Any pathology identified, such as a loose body, posterior tibial edge bony spur, posterior malleolar gutter disorders, FHL tendon impingement, and posterior osteochondral lesions of the talar dome, is easily reached from the proximal portal.

### Postoperative protocol

A compressive bandage was applied, and immediate weightbearing as tolerated with two crutches was recommended. Patients were prescribed plantar flexion and dorsiflexion of the operated ankle, with inversion and eversion of the subtalar joint. At 2 weeks postoperatively, patients were instructed to begin their daily activities and to start swimming and cycling with a high saddle. At 1 month postoperatively, they started using elliptical and stair steppers. Running was planned at 3 postoperative months, with a plan to return to sport-specific training by 4.5 months. The patients were allowed to return to sport when they and their coaches left ready.

## Statistical analysis

Pre- and postoperative AOFAS scores, VAS scores, and Tegner movement scale scores were compared using Student’s* t*-test. Statistical significance was set at *p* < 0.05. Data were analysed using SPSS version 23.0 (SPSS, Chicago, IL).

## Results

Clinical outcomes are recorded in Table 2. The mean age of the patients at last follow-up was 26.3 ± 9.0 (range 15 to 47) years. Of the patients included in the present study, 21 had received an image-guided injection of corticosteroids in the posterior aspect of the ankle at another centre. Another three had received a blind injection of corticosteroids in the posterior aspect of the ankle at another centre. The mean postoperative follow-up was 26.7 ± 12.6 (range 24 to 72) months, while the mean Tegner scale improved from 4.3 ± 0.8 (range 3 to 5) points preoperatively to 9 ± 0.2 points at the last follow-up (*p* < 0.05). The AOFAS scale score improved from a mean of 67.8 ± 6.0 (range 58–76) preoperatively to 96 ± 5.1 (range 87 to 100) at the last follow-up, with 29 of 34 patients (85.3%) reaching the full score of 100 (*p* < 0.05). The time taken to return to full activities of daily living was 8.4 ± 2.1 weeks (range 6 to 11), the time taken to return to sports training was 10.6 ± 3.1 weeks (range 9 to 14), and the time taken to return to sports competition or performance was 14.8 ± 3.9 weeks (range 10 to 19). At the final follow-up, no patient experienced pain, swelling, or tenderness on physical examination; the hyperplantarflexion test was always negative.

**Table 2:** Clinical results (*N* = 34 patients)

	Preoperatively	Postoperatively	*p* value
Tegner scale score	4.3 ± 0.8 (3 to 5)	9 ± 0.2	< 0.05
AOFAS scale score	67.8 ± 6.0 (58 to 76)	96 ± 5.1 (87 to 100)	< 0.05
VAS score	7.8 ± 1.3 (5 to 10)	2.5 ± 0.9 (1 to 4)	< 0.05

No intraoperative adverse events were reported. Furthermore, postoperatively, five patients (14.7%) reported persistent swelling for 2 months. There were no superficial wound infections or venous thromboembolism events. Two patients (5.9%) reported some pain and tenderness by 3 months after the index procedure. In both instances, they had greatly benefitted from the index procedure in the early postoperative phase and had started sport-specific training by 4.5 postoperative months. Both patients were recommended to slow down and to undertake supervised rehabilitation, and they recovered without further intervention in 4 weeks.

## Discussion

The main result of the present study was the favourable clinical and functional outcomes at a mean follow-up of 26.7 months after the endoscopic procedure for the management of PAIS through a double posteromedial technique. PAIS is clinically characterised by posterior ankle pain as result of repetitive or acute forced plantar flexion [22], which has been extensively described in classical ballet dancers [23, 24], in soccer, basketball, and volleyball players, and in runners [25]. If nonoperative management fails to relieve symptoms, surgical excision of the causative impingement is the optimal treatment [14]. Common PAIS management procedures include open excision of the os trigonum through a posterolateral [14, 23, 24] or a posteromedial [26] approach, with a high risk of neurological complications and wound problems due to the open approach itself [26].

The major advantages in the use of an endoscopic compared with an open procedure for the management of PAIS include less tissue damage, a quicker recovery time, and less symptomatic scar formation, all of which are important for athletes [27, 28]. Various endoscopic techniques have been described. One of the most popular is the posterior hindfoot endoscopy described by van Dijk et al. [29], who reported one case of arthroscopic management of PAIS due to a symptomatic os trigonum, with excellent results achieved through a posteromedial and a posterolateral portal. The posterior approach with the patient prone and with two para-Achilles tendon portals, one medial and one lateral, has been in use for nearly two decades, and it has been shown to be safe and effective. Some authors have expressed concern about portal placement close to the posterior tibial neurovascular bundle and the effect of ankle dorsiflexion during endoscopy [30]. When the procedure is performed with the patient prone and using a single posteromedial and a single posterolateral portal, both immediately adjacent to the Achilles tendon, and with the ankle held at a neutral–neutral position with portals described by van Dijk, the greatest margin of safety from neurovascular structures is achieved. This is what was accomplished when we produced the distal posteromedial portal. As shown in our previous studies on the use of the double posteromedial portal, the proximal posteromedial portal is 45–50 mm proximal to the distal one and well in the safe area [16]. Indeed, in our setting, we have routinely used this approach for the past 15 years and have never encountered any neurovascular compromise.

The approach used in the present study is undertaken with the patient supine and involves two posteromedial para-Achilles tendon entry portals [31]. To our knowledge, this is the only investigation in which this technique was used systematically to approach the pathology at hand. The double posteromedial approach with the patient supine has several advantages. For example, positioning the patient is much easier, as the patient is supine instead of prone. Hence, if general anaesthesia is used, the patient needs to be intubated to secure the airways. Triangulation of the arthroscope and the working instrument is easily performed, and we have experienced no technical issues in undertaking the desired interventions.

The double posteromedial portal approach used in the present study [31] is safe and allows excellent vision of the posterior compartment without any neurovascular or tendon complications [16]. In addition, the patient is supine, and therefore the operating theatre setup is simpler and achieved faster than when positioning the patient prone. Also, monitoring is easier with the patient supine.

The mean time of return to sports was 10.6 ± 3.1 weeks (range 9 to 14), which compares favourably to published literature on a military population [18] and is better than for dancers [32]. Similar clinical outcomes were found for open and arthroscopic excision of a symptomatic os trigonum in a 41-case series [14], but, regarding the different complication rates, it was reported that the overall complication rate after endoscopic management was 4.8% (25 of 521 cases), with a neurological complication rate of 3.6% (19 of 521 cases), while the overall complication rate for open surgery was up to 14.7% [17]. In another relatively recent systematic review, the reported complication rate was 15.9% (23 cases) for open surgery and 7.3% (20 cases) for endoscopic surgery [33]. On the other hand, Nickisch et al. [34] found a complication rate of 8.5% in 186 patients managed with two-portal posterior ankle arthroscopy. This higher complication rate likely resulted from the population heterogeneity and a lack of differentiation between posterior ankle and hindfoot arthroscopy and endoscopy. Furthermore, Ribbans et al. reported an 80% rate of return to the pre-injury level of sport in both endoscopic and open surgery groups at an average of 8.9 weeks and 14.8 weeks, respectively [17].

Our study reported five patients (14.7%) with persistent swelling for 2 months and two patients (5.9%) with pain and tenderness for 3 months postoperatively, but no patient developed a superficial wound infection or venous thromboembolism. Our complications relate to findings present in almost any orthopaedic procedure in the lower limb and constitute minor issues that resolved spontaneously over time.

Jerosch [15] described the results of arthroscopic resection of a symptomatic os trigonum by two posterior portals in 10 patients, and Ahn et al. [35] compared the results of arthroscopic and endoscopic management of PAIS due to os trigonum, showing that both procedures were effective and safe [35]. However, they reported a failure rate of 12.5% in patients with a large os trigonum who underwent endoscopic excision [35].

In the present study, at last follow-up, none of the patients experienced pain during plantar flexion and all were able to return to sports with a good level of performance. To the best of our knowledge, this study reports one of the largest case series of athletes who were managed with a posterior ankle endoscopic technique for PAIS, and it is the first one where the procedure was performed with the patient supine using the double posteromedial portals. The major limitation of the present study was the absence of a control group: ideally, an appropriately powered randomised controlled trial where the traditional lateral and medial posterior para-Achilles portals are used with the patient prone would have been compared to the present approach with the patient supine and using the double posteromedial portal. This is the first study that reports results obtained using two posteromedial portals to manage PAIS in athletic patients and not only dancers or military personnel who underwent surgery. The low complication rate and relatively simple complications reported may be related to these two posteromedial portals, suggesting that being less aggressive on soft tissues could reduce adverse events such as haematoma.

## Conclusions

Our study demonstrated that posterior endoscopy for the management of PAIS using the double posteromedial portal technique with the patient supine is safe and characterised by excellent results with low morbidity. These results make this approach attractive for athletes who wish to return to their full preoperative activity level and whose sport involves in repeated forced plantarflexed position of the ankle.

## Data Availability

The data underlying this article are available in the article and in its online supplementary material.

## Abbreviations

PAIS: Posterior ankle impingement syndrome
FHL: Flexor hallucis longus
AOFAS: American Orthopaedic Foot and Ankle Society

## References

[1] Maquirriain J (2005). Posterior ankle impingement syndrome. J Am Acad Orthop Surg.

[2] Howse AJ (1982). Posterior block of the ankle joint in dancers. Foot Ankle.

[3] Brodsky AE, Khalil MA (1987). Talar compression syndrome. Foot Ankle.

[4] Chao W (2004). Os trigonum. Foot Ankle Clin.

[5] Moser BR (2011). Posterior ankle impingement in the dancer. Curr Sports Med Rep.

[6] Russell JA, Kruse DW, Koutedakis Y, Wyon MA (2012) Pathoanatomy of anterior ankle impingement in dancers. J Dance Med Sci 16:101–108 26730938

[7] Wiegerinck JI, Vroemen JC, van Dongen TH et al (2014) The posterior impingement view: an alternative conventional projection to detect bony posterior ankle impingement. Arthroscopy 30:1311–1316. 10.1016/j.arthro.2014.05.006 10.1016/j.arthro.2014.05.006 25023737

[8] McCarthy CL, Wilson DJ, Coltman TP (2008). Anterolateral ankle impingement: findings and diagnostic accuracy with ultrasound imaging. Skeletal Radiol.

[9] Pesquer L, Guillo S, Meyer P, Hauger O (2014). US in ankle impingement syndrome. J Ultrasound.

[10] Jose J, Mirpuri T, Lesniak B, Kaplan L (2014). Sonographically guided therapeutic injections in the meniscoid lesion in patients with anteromedial ankle impingement syndrome. Foot Ankle Spec.

[11] Robinson P, Bollen SR (2006). Posterior ankle impingement in professional soccer players: effectiveness of sonographically guided therapy. Am J Roentgenol.

[12] Russell JA, Kruse DW, Koutedakis Y (2010). Pathoanatomy of posterior ankle impingement in ballet dancers. Clin Anat N Y N.

[13] Lavery KP, McHale KJ, Rossy WH, Theodore G (2016). Ankle impingement. J Orthop Surg.

[14] Abramowitz Y, Wollstein R, Barzilay Y (2003). Outcome of resection of a symptomatic os trigonum. J Bone Joint Surg Am.

[15] Jerosch J (1998) Subtalar arthroscopy—indications and surgical technique. Knee Surg Sports Traumatol Arthrosc 6:122–128. 10.1007/s001670050084 10.1007/s001670050084 9604198

[16] Allegra F, Bonacci E, El Boustany S (2016). Endoscopy of the posterior aspect of the ankle: double posteromedial portals. Sports Med Arthrosc Rev.

[17] Ribbans WJ, Ribbans HA, Cruickshank JA, Wood EV (2015) The management of posterior ankle impingement syndrome in sport: a review. Foot Ankle Surg 21:1–10. 10.1016/j.fas.2014.08.006 10.1016/j.fas.2014.08.006 25682399

[18] Georgiannos D, Bisbinas I (2017). Endoscopic versus open excision of os trigonum for the treatment of posterior ankle impingement syndrome in an athletic population: a randomized controlled study with 5-year follow-up. Am J Sports Med.

[19] Leigheb M, Janicka P, Andorno S (2016). Italian translation, cultural adaptation and validation of the “American Orthopaedic Foot and Ankle Society’s (AOFAS) ankle-hindfoot scale”. Acta Bio-Medica Atenei Parm.

[20] Halasi T, Kynsburg A, Tállay A, Berkes I (2004). Development of a new activity score for the evaluation of ankle instability. Am J Sports Med.

[21] Heller GZ, Manuguerra M, Chow R (2016) How to analyze the Visual Analogue Scale: myths, truths and clinical relevance. Scand J Pain 13:67–75. 10.1016/j.sjpain.2016.06.012 10.1016/j.sjpain.2016.06.012 28850536

[22] Hedrick MR, McBryde AM (1994). Posterior ankle impingement. Foot Ankle Int.

[23] Hamilton WG, Geppert MJ, Thompson FM (1996). Pain in the posterior aspect of the ankle in dancers. Differential diagnosis and operative treatment. J Bone Joint Surg Am.

[24] Marotta JJ, Micheli LJ (1992). Os trigonum impingement in dancers. Am J Sports Med.

[25] Tey M, Monllau JC, Centenera JM, Pelfort X (2007) Benefits of arthroscopic tuberculoplasty in posterior ankle impingement syndrome. Knee Surg Sports Traumatol Arthrosc 15:1235–1239. 10.1007/s00167-007-0349-1 10.1007/s00167-007-0349-1 17589829

[26] Wredmark T, Carlstedt CA, Bauer H, Saartok T (1991). Os trigonum syndrome: a clinical entity in ballet dancers. Foot Ankle.

[27] Zwiers R, Wiegerinck JI, Murawski CD et al (2013) Surgical treatment for posterior ankle impingement. Arthrosc J Arthrosc Relat Surg 29:1263–1270. 10.1016/j.arthro.2013.01.029 10.1016/j.arthro.2013.01.029 23541613

[28] Willits K, Sonneveld H, Amendola A et al (2008) Outcome of posterior ankle arthroscopy for hindfoot impingement. Arthrosc J Arthrosc Relat Surg 24:196–202. 10.1016/j.arthro.2007.08.025 10.1016/j.arthro.2007.08.025 18237704

[29] van Dijk CN (2006). Hindfoot endoscopy. Foot Ankle Clin.

[30] Urgüden M, Cevikol C, Dabak TK et al (2009) Effect of joint motion on safety of portals in posterior ankle arthroscopy. Arthrosc J Arthrosc Relat Surg 25:1442–1446. 10.1016/j.arthro.2009.05.004 10.1016/j.arthro.2009.05.004 19962072

[31] van Dijk CN, Scholten PE, Krips R (2000) A 2-portal endoscopic approach for diagnosis and treatment of posterior ankle pathology. Arthrosc J Arthrosc Relat Surg 16:871–876. 10.1053/jars.2000.19430 10.1053/jars.2000.19430 11078550

[32] Rietveld ABMB, Hagemans FMT, Haitjema S et al (2018) Results of treatment of posterior ankle impingement syndrome and flexor hallucis longus tendinopathy in dancers: a systematic review. J Dance Med Sci. 22:19–32. 10.12678/1089-313X.22.1.19 10.12678/1089-313X.22.1.19 29510786

[33] Yilmaz C, Eskandari MM (2006) Arthroscopic excision of the talar Stieda’s process. Arthrosc J Arthrosc Relat Surg 22:225.e1-225.e3. 10.1016/j.arthro.2005.11.004 10.1016/j.arthro.2005.11.004 16458810

[34] Nickisch F, Barg A, Saltzman CL (2012). Postoperative complications of posterior ankle and hindfoot arthroscopy. J Bone Joint Surg Am.

[35] Ahn JH, Kim Y-C, Kim H-Y (2013). Arthroscopic versus posterior endoscopic excision of a symptomatic os trigonum: a retrospective cohort study. Am J Sports Med.

